# Crystal structure of 4-(2-azido­phen­yl)-5-benzoyl-2-(1*H*-indol-3-yl)-1*H*-pyrrole-3-carbo­nitrile

**DOI:** 10.1107/S2056989015006921

**Published:** 2015-04-22

**Authors:** G. Vimala, J. Kamal Raja, Y. Amina Naaz, P. T. Preumal, A. SubbiahPandi

**Affiliations:** a Department of Physics, Presidency College (Autonomous), Chennai 600 005, India; b Organic Chemistry Division, Central Leather Research Institute (CSIR), Adyar, Chennai 600 020, India

**Keywords:** crystal structure, indole derivatives, pyrrole-3-carbo­nitrile, hydrogen bonding

## Abstract

In the title compound, C_26_H_16_N_6_O, the dihedral angles between the central pyrrole ring and the pendant indole ring system (r.m.s. deviation = 0.027 Å) and the azide-bearing benzene ring are 37.56 (8) and 51.62 (11)°, respectively. The azide group is almost coplanar with its attached benzene ring [C—C—N—N = 3.8 (3)°]. The benzoyl benzene ring is disordered over two orientations twisted with respect to each other by 9.29 (8)° in a 0.514 (2):0.486 (2) ratio. In the crystal, inversion dimers linked by pairs of N_p_—H⋯O (p = pyrrole) hydrogen bonds generate *R*_2_^2^(10) loops. A second inversion dimer arises from a pair of N_i_—H⋯N_c_ (i = indole and c = cyanide) hydrogen bonds, which generates an *R*_2_^2^(16) loop. Together, the hydrogen bonds lead to [011] chains in the crystal.

## Related literature   

For background to indole derivatives, see: Srivastava, Anupam & Pandeya (2011 ▸). For related structures, see: Srinivasan *et al.* (2012 ▸); Inglebert *et al.* (2013 ▸).
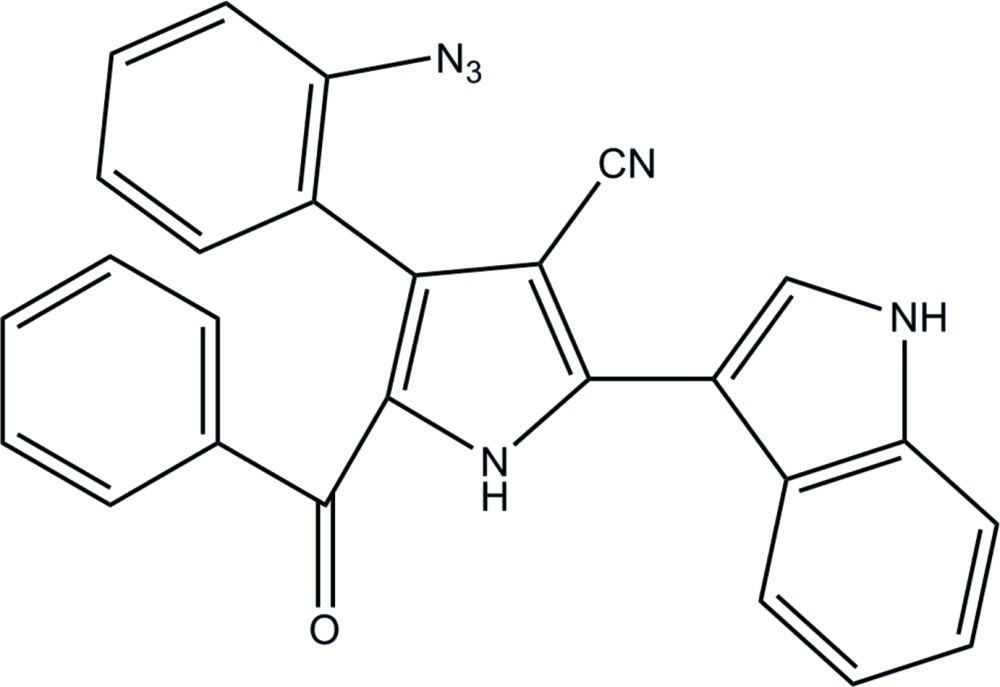


## Experimental   

### Crystal data  

C_26_H_16_N_6_O
*M* *_r_* = 428.45
Triclinic, 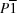

*a* = 8.1834 (6) Å
*b* = 11.3713 (8) Å
*c* = 12.6853 (8) Å
α = 108.070 (3)°
β = 105.164 (4)°
γ = 90.256 (3)°
*V* = 1078.42 (13) Å^3^
*Z* = 2
Mo *K*α radiation
μ = 0.09 mm^−1^
*T* = 293 K
0.35 × 0.20 × 0.15 mm

### Data collection  

Bruker Kappa APEXII CCD diffractometer
Absorption correction: multi-scan (*SADABS*; Bruker, 2004 ▸) *T*_min_ = 0.901, *T*_max_ = 0.987
20625 measured reflections
4315 independent reflections
3062 reflections with *I* > 2σ(*I*)
*R*_int_ = 0.033

### Refinement  

*R*[*F* ^2^ > 2σ(*F* ^2^)] = 0.046
*wR*(*F* ^2^) = 0.143
*S* = 1.04
4315 reflections
325 parameters
2 restraints
H atoms treated by a mixture of independent and constrained refinement
Δρ_max_ = 0.21 e Å^−3^
Δρ_min_ = −0.25 e Å^−3^

### 

Data collection: *APEX2* (Bruker, 2004 ▸); cell refinement: *SAINT* (Bruker, 2004 ▸); data reduction: *SAINT*; program(s) used to solve structure: *SHELXS97* (Sheldrick, 2008 ▸); program(s) used to refine structure: *SHELXL97* (Sheldrick, 2008 ▸); molecular graphics: *ORTEP-3 for Windows* (Farrugia, 2012 ▸); software used to prepare material for publication: *PLATON* (Spek, 2009 ▸).

## Supplementary Material

Crystal structure: contains datablock(s) global, I. DOI: 10.1107/S2056989015006921/hb7394sup1.cif

Structure factors: contains datablock(s) I. DOI: 10.1107/S2056989015006921/hb7394Isup2.hkl

Supporting information file. DOI: 10.1107/S2056989015006921/hb7394Isup3.cml

**Fig. 1.**
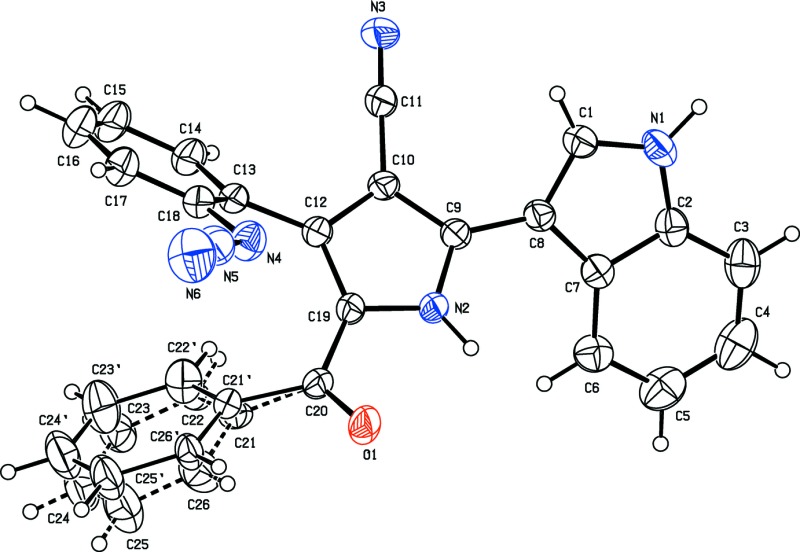
The molecular structure of the title compound, with displacement ellipsoids drawn at 30% probability level.

**Fig. 2.**
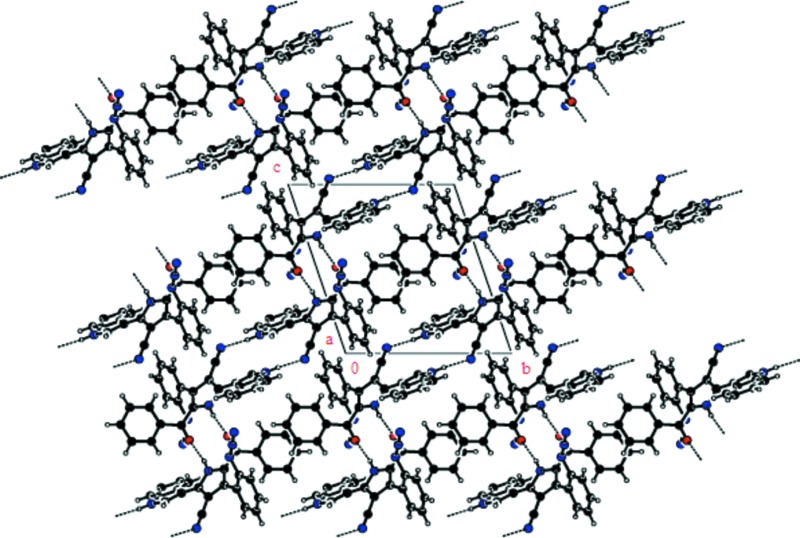
The crystal packing of the title compound viewed along c axis. Hydrogen atoms are omitted for clarity.

. DOI: 10.1107/S2056989015006921/hb7394fig1.tif
The mol­ecular structure of the title compound, with displacement ellipsoids drawn at 30% probability level.

c . DOI: 10.1107/S2056989015006921/hb7394fig2.tif
The crystal packing of the title compound viewed along *c* axis. Hydrogen atoms are omitted for clarity.

CCDC reference: 933255

Additional supporting information:  crystallographic information; 3D view; checkCIF report

## Figures and Tables

**Table 1:** Hydrogen-bond geometry (, )

*D*H*A*	*D*H	H*A*	*D* *A*	*D*H*A*
N2H2*A*O1^i^	0.92(1)	1.95(1)	2.8526(17)	166(2)
N1H1*A*N3^ii^	0.92(1)	2.10(1)	2.988(2)	163(2)

Symmetry codes: (i) 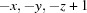; (ii) 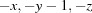.

## supplementary crystallographic information

### S1. Experimental

To a stirred mixture of 2-azido aldehydes 1 (1.0 mmol), 2- (1*H*-3-indolylcarbonyl)-3-aryl-2-propenenitriles 2 (1.0 mmol) and phenacylazides 3 (1.0 mmol) in water (3 ml) piperidine (0.25 mmol) was added at 80 ^0^ C. The turbid solution slowly turned into a clear solution followed by the formation of solid with 0.75 min. After completion of the reaction as indicated by thin layer chromatography (TLC), the solid was filtered and washed with pet-ether: EtOAc mixture (1: 1 ratio *v*/*v*, 5 ml) to give pure compounds. The compound was recrystallized from methanol to yield yellow crystals. The yield of the isolated product was 91%. Yellow blocks were obtained by slow evaporation of a solution of the title compound in methanol at room temperature.

### S2. Refinement

All H atoms were fixed geometrically and allowed to ride on their parent C atoms, with C—H distances fixed in the range 0.93–0.97 Å with *U*_iso_(H) = 1.5*U*_eq_(C) for methyl H atoms and 1.2*U*_eq_(C) for all other H atoms.

### Crystal data

**Table:**

C_26_H_16_N_6_O	*Z* = 2
*M**_r_* = 428.45	*F*(000) = 444
Triclinic, *P*1	*D*_x_ = 1.319 Mg m^−^^3^
Hall symbol: -P 1	Mo *K*α radiation, λ = 0.71073 Å
*a* = 8.1834 (6) Å	Cell parameters from 5359 reflections
*b* = 11.3713 (8) Å	θ = 2.6–24.5°
*c* = 12.6853 (8) Å	µ = 0.09 mm^−^^1^
α = 108.070 (3)°	*T* = 293 K
β = 105.164 (4)°	Block, yellow
γ = 90.256 (3)°	0.35 × 0.20 × 0.15 mm
*V* = 1078.42 (13) Å^3^	

### Data collection

**Table:**

Bruker Kappa APEXII CCD diffractometer	4315 independent reflections
Radiation source: fine-focus sealed tube	3062 reflections with *I* > 2σ(*I*)
Graphite monochromator	*R*_int_ = 0.033
ω and φ scan	θ_max_ = 26.2°, θ_min_ = 2.1°
Absorption correction: multi-scan (*SADABS*; Bruker, 2004)	*h* = −10→10
*T*_min_ = 0.901, *T*_max_ = 0.987	*k* = −14→14
20625 measured reflections	*l* = −15→11

### Refinement

**Table:**

Refinement on *F*^2^	Primary atom site location: structure-invariant direct methods
Least-squares matrix: full	Secondary atom site location: difference Fourier map
*R*[*F*^2^ > 2σ(*F*^2^)] = 0.046	Hydrogen site location: inferred from neighbouring sites
*wR*(*F*^2^) = 0.143	H atoms treated by a mixture of independent and constrained refinement
*S* = 1.04	*w* = 1/[σ^2^(*F*_o_^2^) + (0.0727*P*)^2^ + 0.1697*P*] where *P* = (*F*_o_^2^ + 2*F*_c_^2^)/3
4315 reflections	(Δ/σ)_max_ < 0.001
325 parameters	Δρ_max_ = 0.21 e Å^−^^3^
2 restraints	Δρ_min_ = −0.25 e Å^−^^3^

### Special details

**Table:**

Geometry. All e.s.d.'s (except the e.s.d. in the dihedral angle between two l.s. planes) are estimated using the full covariance matrix. The cell e.s.d.'s are taken into account individually in the estimation of e.s.d.'s in distances, angles and torsion angles; correlations between e.s.d.'s in cell parameters are only used when they are defined by crystal symmetry. An approximate (isotropic) treatment of cell e.s.d.'s is used for estimating e.s.d.'s involving l.s. planes.
Refinement. Refinement of *F*^2^ against ALL reflections. The weighted *R*-factor *wR* and goodness of fit *S* are based on *F*^2^, conventional *R*-factors *R* are based on *F*, with *F* set to zero for negative *F*^2^. The threshold expression of *F*^2^ > σ(*F*^2^) is used only for calculating *R*-factors(gt) *etc*. and is not relevant to the choice of reflections for refinement. *R*-factors based on *F*^2^ are statistically about twice as large as those based on *F*, and *R*- factors based on ALL data will be even larger.

### Fractional atomic coordinates and isotropic or equivalent isotropic displacement parameters (Å^2^)

**Table:**

	*x*	*y*	*z*	*U*_iso_*/*U*_eq_	Occ. (<1)
C1	−0.0917 (3)	−0.39241 (16)	0.12464 (15)	0.0547 (5)	
H1	0.0075	−0.4097	0.1024	0.066*	
C2	−0.3428 (2)	−0.42040 (17)	0.15320 (15)	0.0543 (5)	
C3	−0.4944 (3)	−0.4692 (2)	0.15954 (19)	0.0750 (6)	
H3	−0.5245	−0.5544	0.1333	0.090*	
C4	−0.5968 (3)	−0.3873 (3)	0.2057 (2)	0.0886 (8)	
H4	−0.6987	−0.4173	0.2117	0.106*	
C5	−0.5540 (3)	−0.2602 (3)	0.2443 (2)	0.0820 (7)	
H5	−0.6281	−0.2069	0.2747	0.098*	
C6	−0.4056 (3)	−0.2119 (2)	0.23854 (17)	0.0621 (5)	
H6	−0.3783	−0.1264	0.2641	0.075*	
C7	−0.2954 (2)	−0.29262 (16)	0.19367 (14)	0.0473 (4)	
C8	−0.1326 (2)	−0.27646 (15)	0.17555 (13)	0.0454 (4)	
C9	−0.0284 (2)	−0.16159 (14)	0.20815 (13)	0.0431 (4)	
C10	0.0785 (2)	−0.12214 (15)	0.15429 (13)	0.0451 (4)	
C11	0.1096 (2)	−0.19675 (16)	0.05005 (15)	0.0515 (4)	
C12	0.1570 (2)	−0.00185 (15)	0.22359 (14)	0.0449 (4)	
C13	0.2907 (2)	0.06488 (15)	0.20018 (15)	0.0504 (4)	
C14	0.2675 (3)	0.07898 (19)	0.09230 (18)	0.0664 (5)	
H14	0.1646	0.0494	0.0362	0.080*	
C15	0.3950 (4)	0.1362 (2)	0.0672 (2)	0.0847 (7)	
H15	0.3775	0.1455	−0.0052	0.102*	
C16	0.5461 (4)	0.1790 (2)	0.1486 (3)	0.0894 (8)	
H16	0.6317	0.2173	0.1314	0.107*	
C17	0.5740 (3)	0.1663 (2)	0.2556 (2)	0.0759 (6)	
H17	0.6777	0.1965	0.3107	0.091*	
C18	0.4479 (3)	0.10869 (17)	0.28161 (17)	0.0561 (5)	
C19	0.0923 (2)	0.03026 (15)	0.31786 (14)	0.0447 (4)	
C20	0.1247 (2)	0.13755 (16)	0.42355 (14)	0.0476 (4)	
N1	−0.2164 (2)	−0.47857 (14)	0.11122 (14)	0.0612 (4)	
N2	−0.01795 (18)	−0.06776 (12)	0.30625 (11)	0.0453 (3)	
N3	0.1341 (2)	−0.25813 (15)	−0.03298 (14)	0.0697 (5)	
N4	0.4701 (2)	0.08726 (16)	0.38885 (14)	0.0642 (4)	
N5	0.6075 (2)	0.12980 (17)	0.46065 (17)	0.0697 (5)	
O1	0.1155 (2)	0.12110 (12)	0.51215 (10)	0.0720 (4)	
N6	0.7264 (2)	0.16569 (9)	0.53511 (10)	0.1037 (7)	
C21	0.1467 (2)	0.26296 (9)	0.42353 (10)	0.0457 (16)	0.486 (6)
C22	0.0825 (2)	0.29348 (9)	0.32352 (10)	0.0454 (13)	0.486 (6)
H22	0.0316	0.2315	0.2542	0.054*	0.486 (6)
C23	0.0943 (2)	0.41672 (9)	0.32710 (10)	0.0697 (16)	0.486 (6)
H23	0.0513	0.4371	0.2602	0.084*	0.486 (6)
C24	0.1703 (2)	0.50943 (9)	0.43068 (10)	0.094 (2)	0.486 (6)
H24	0.1781	0.5919	0.4331	0.113*	0.486 (6)
C25	0.2345 (2)	0.47891 (9)	0.53069 (10)	0.104 (2)	0.486 (6)
H25	0.2853	0.5409	0.6000	0.125*	0.486 (6)
C26	0.2227 (2)	0.35568 (9)	0.52712 (10)	0.086 (2)	0.486 (6)
H26	0.2657	0.3353	0.5940	0.103*	0.486 (6)
C21'	0.1727 (2)	0.26171 (9)	0.41748 (10)	0.0465 (16)	0.514 (6)
C22'	0.1178 (2)	0.29254 (9)	0.31689 (10)	0.0723 (18)	0.514 (6)
H22'	0.0559	0.2330	0.2489	0.087*	0.514 (6)
C23'	0.1552 (2)	0.41233 (9)	0.31797 (10)	0.095 (2)	0.514 (6)
H23'	0.1185	0.4330	0.2507	0.114*	0.514 (6)
C24'	0.2476 (2)	0.50129 (9)	0.41964 (10)	0.095 (2)	0.514 (6)
H24'	0.2727	0.5814	0.4204	0.114*	0.514 (6)
C25'	0.3026 (2)	0.47046 (9)	0.52023 (10)	0.0780 (17)	0.514 (6)
H25'	0.3644	0.5300	0.5883	0.094*	0.514 (6)
C26'	0.2651 (2)	0.35067 (9)	0.51915 (10)	0.0523 (12)	0.514 (6)
H26'	0.3019	0.3300	0.5865	0.063*	0.514 (6)
H2A	−0.060 (2)	−0.0740 (17)	0.3647 (12)	0.059 (5)*	
H1A	−0.214 (3)	−0.5632 (10)	0.0821 (18)	0.082 (7)*	

### Atomic displacement parameters (Å^2^)

**Table:**

	*U*^11^	*U*^22^	*U*^33^	*U*^12^	*U*^13^	*U*^23^
C1	0.0648 (12)	0.0460 (10)	0.0521 (10)	0.0055 (9)	0.0199 (9)	0.0112 (8)
C2	0.0572 (11)	0.0520 (10)	0.0468 (10)	−0.0059 (9)	0.0027 (8)	0.0162 (8)
C3	0.0660 (14)	0.0772 (15)	0.0734 (14)	−0.0191 (12)	−0.0016 (11)	0.0303 (12)
C4	0.0491 (13)	0.128 (2)	0.0960 (18)	−0.0048 (15)	0.0107 (12)	0.0547 (17)
C5	0.0555 (13)	0.110 (2)	0.0913 (17)	0.0225 (13)	0.0218 (12)	0.0458 (15)
C6	0.0607 (12)	0.0651 (12)	0.0627 (12)	0.0152 (10)	0.0148 (10)	0.0251 (10)
C7	0.0530 (10)	0.0472 (10)	0.0397 (9)	0.0038 (8)	0.0070 (7)	0.0160 (7)
C8	0.0558 (10)	0.0413 (9)	0.0378 (8)	0.0030 (8)	0.0124 (7)	0.0115 (7)
C9	0.0537 (10)	0.0392 (9)	0.0381 (8)	0.0053 (7)	0.0144 (7)	0.0132 (7)
C10	0.0586 (10)	0.0421 (9)	0.0391 (8)	0.0082 (8)	0.0204 (8)	0.0136 (7)
C11	0.0669 (12)	0.0448 (9)	0.0489 (10)	0.0070 (8)	0.0234 (9)	0.0175 (8)
C12	0.0570 (10)	0.0413 (9)	0.0423 (9)	0.0072 (8)	0.0197 (8)	0.0165 (7)
C13	0.0664 (12)	0.0412 (9)	0.0556 (10)	0.0095 (8)	0.0334 (9)	0.0185 (8)
C14	0.0871 (15)	0.0662 (12)	0.0653 (12)	0.0134 (11)	0.0384 (11)	0.0336 (10)
C15	0.116 (2)	0.0852 (16)	0.0867 (17)	0.0164 (15)	0.0601 (17)	0.0484 (14)
C16	0.099 (2)	0.0833 (17)	0.119 (2)	0.0062 (15)	0.0690 (18)	0.0466 (16)
C17	0.0720 (14)	0.0661 (13)	0.1031 (18)	0.0009 (11)	0.0456 (13)	0.0283 (13)
C18	0.0648 (12)	0.0458 (10)	0.0679 (12)	0.0084 (9)	0.0336 (10)	0.0199 (9)
C19	0.0577 (10)	0.0397 (9)	0.0411 (9)	0.0007 (8)	0.0196 (8)	0.0143 (7)
C20	0.0571 (10)	0.0467 (10)	0.0415 (9)	−0.0014 (8)	0.0220 (8)	0.0109 (7)
N1	0.0754 (11)	0.0384 (9)	0.0607 (10)	−0.0011 (8)	0.0138 (8)	0.0075 (7)
N2	0.0604 (9)	0.0415 (8)	0.0373 (7)	−0.0008 (6)	0.0205 (7)	0.0115 (6)
N3	0.1019 (14)	0.0555 (10)	0.0578 (10)	0.0102 (9)	0.0425 (10)	0.0099 (8)
N4	0.0575 (10)	0.0688 (11)	0.0639 (10)	−0.0039 (8)	0.0140 (8)	0.0207 (9)
N5	0.0620 (11)	0.0653 (11)	0.0799 (13)	0.0065 (9)	0.0191 (10)	0.0210 (10)
O1	0.1137 (12)	0.0603 (8)	0.0442 (7)	−0.0204 (8)	0.0357 (7)	0.0086 (6)
N6	0.0723 (14)	0.1120 (18)	0.1114 (18)	−0.0078 (13)	0.0005 (13)	0.0353 (15)
C21	0.049 (3)	0.048 (4)	0.039 (3)	0.005 (2)	0.020 (3)	0.006 (3)
C22	0.058 (2)	0.038 (3)	0.042 (3)	0.012 (2)	0.019 (2)	0.012 (2)
C23	0.098 (4)	0.053 (3)	0.062 (3)	0.010 (2)	0.020 (3)	0.025 (3)
C24	0.137 (6)	0.051 (3)	0.109 (5)	0.004 (3)	0.044 (4)	0.036 (3)
C25	0.129 (6)	0.061 (4)	0.105 (5)	−0.017 (3)	0.019 (4)	0.017 (4)
C26	0.107 (4)	0.066 (5)	0.076 (5)	0.003 (3)	0.021 (4)	0.016 (4)
C21'	0.055 (3)	0.040 (3)	0.053 (4)	0.002 (2)	0.028 (3)	0.016 (3)
C22'	0.077 (3)	0.069 (4)	0.070 (4)	−0.005 (3)	0.014 (3)	0.027 (3)
C23'	0.115 (4)	0.072 (4)	0.108 (5)	0.005 (3)	0.020 (4)	0.052 (4)
C24'	0.149 (6)	0.046 (3)	0.090 (4)	−0.008 (3)	0.034 (4)	0.022 (3)
C25'	0.113 (4)	0.044 (3)	0.065 (3)	−0.018 (2)	0.016 (3)	0.008 (2)
C26'	0.075 (3)	0.037 (3)	0.039 (3)	−0.012 (2)	0.012 (2)	0.008 (2)

### Geometric parameters (Å, º)

**Table:**

C1—N1	1.351 (2)	C18—N4	1.421 (2)
C1—C8	1.362 (2)	C19—N2	1.378 (2)
C1—H1	0.9300	C19—C20	1.467 (2)
C2—N1	1.367 (3)	C20—O1	1.2158 (19)
C2—C3	1.388 (3)	C20—C21	1.4368 (19)
C2—C7	1.398 (2)	C20—C21'	1.4940 (19)
C3—C4	1.356 (4)	N1—H1A	0.920 (10)
C3—H3	0.9300	N2—H2A	0.916 (9)
C4—C5	1.384 (4)	N4—N5	1.231 (2)
C4—H4	0.9300	N5—N6	1.132 (2)
C5—C6	1.359 (3)	C21—C22	1.3900
C5—H5	0.9300	C21—C26	1.3900
C6—C7	1.390 (3)	C22—C23	1.3900
C6—H6	0.9300	C22—H22	0.9300
C7—C8	1.430 (2)	C23—C24	1.3900
C8—C9	1.441 (2)	C23—H23	0.9300
C9—N2	1.347 (2)	C24—C25	1.3900
C9—C10	1.390 (2)	C24—H24	0.9300
C10—C12	1.417 (2)	C25—C26	1.3900
C10—C11	1.419 (2)	C25—H25	0.9300
C11—N3	1.137 (2)	C26—H26	0.9300
C12—C19	1.381 (2)	C21'—C22'	1.3900
C12—C13	1.471 (2)	C21'—C26'	1.3900
C13—C14	1.391 (3)	C22'—C23'	1.3900
C13—C18	1.396 (3)	C22'—H22'	0.9300
C14—C15	1.382 (3)	C23'—C24'	1.3900
C14—H14	0.9300	C23'—H23'	0.9300
C15—C16	1.359 (4)	C24'—C25'	1.3900
C15—H15	0.9300	C24'—H24'	0.9300
C16—C17	1.369 (4)	C25'—C26'	1.3900
C16—H16	0.9300	C25'—H25'	0.9300
C17—C18	1.382 (3)	C26'—H26'	0.9300
C17—H17	0.9300		
			
N1—C1—C8	110.10 (17)	N2—C19—C20	117.84 (14)
N1—C1—H1	124.9	C12—C19—C20	134.10 (15)
C8—C1—H1	124.9	O1—C20—C21	118.38 (15)
N1—C2—C3	130.43 (19)	O1—C20—C19	118.84 (15)
N1—C2—C7	107.62 (16)	C21—C20—C19	122.36 (14)
C3—C2—C7	121.9 (2)	O1—C20—C21'	123.14 (15)
C4—C3—C2	117.1 (2)	C21—C20—C21'	9.3
C4—C3—H3	121.4	C19—C20—C21'	117.99 (13)
C2—C3—H3	121.4	C1—N1—C2	109.22 (15)
C3—C4—C5	121.9 (2)	C1—N1—H1A	125.6 (14)
C3—C4—H4	119.0	C2—N1—H1A	125.1 (14)
C5—C4—H4	119.0	C9—N2—C19	111.13 (13)
C6—C5—C4	121.2 (2)	C9—N2—H2A	124.4 (12)
C6—C5—H5	119.4	C19—N2—H2A	123.3 (12)
C4—C5—H5	119.4	N5—N4—C18	115.33 (17)
C5—C6—C7	118.7 (2)	N6—N5—N4	172.4 (2)
C5—C6—H6	120.6	C22—C21—C26	120.0
C7—C6—H6	120.6	C22—C21—C20	120.87 (8)
C6—C7—C2	119.01 (17)	C26—C21—C20	118.97 (8)
C6—C7—C8	134.25 (17)	C21—C22—C23	120.0
C2—C7—C8	106.73 (15)	C21—C22—H22	120.0
C1—C8—C7	106.32 (15)	C23—C22—H22	120.0
C1—C8—C9	126.31 (16)	C24—C23—C22	120.0
C7—C8—C9	127.33 (15)	C24—C23—H23	120.0
N2—C9—C10	106.05 (14)	C22—C23—H23	120.0
N2—C9—C8	122.71 (14)	C25—C24—C23	120.0
C10—C9—C8	131.23 (15)	C25—C24—H24	120.0
C9—C10—C12	109.26 (13)	C23—C24—H24	120.0
C9—C10—C11	123.89 (15)	C24—C25—C26	120.0
C12—C10—C11	126.73 (15)	C24—C25—H25	120.0
N3—C11—C10	178.92 (19)	C26—C25—H25	120.0
C19—C12—C10	105.61 (14)	C25—C26—C21	120.0
C19—C12—C13	129.39 (16)	C25—C26—H26	120.0
C10—C12—C13	124.77 (14)	C21—C26—H26	120.0
C14—C13—C18	117.82 (17)	C22'—C21'—C26'	120.0
C14—C13—C12	120.37 (18)	C22'—C21'—C20	122.25 (8)
C18—C13—C12	121.68 (15)	C26'—C21'—C20	117.58 (8)
C15—C14—C13	121.1 (2)	C23'—C22'—C21'	120.0
C15—C14—H14	119.5	C23'—C22'—H22'	120.0
C13—C14—H14	119.5	C21'—C22'—H22'	120.0
C16—C15—C14	119.8 (2)	C22'—C23'—C24'	120.0
C16—C15—H15	120.1	C22'—C23'—H23'	120.0
C14—C15—H15	120.1	C24'—C23'—H23'	120.0
C15—C16—C17	120.9 (2)	C25'—C24'—C23'	120.0
C15—C16—H16	119.6	C25'—C24'—H24'	120.0
C17—C16—H16	119.6	C23'—C24'—H24'	120.0
C16—C17—C18	119.9 (2)	C24'—C25'—C26'	120.0
C16—C17—H17	120.0	C24'—C25'—H25'	120.0
C18—C17—H17	120.0	C26'—C25'—H25'	120.0
C17—C18—C13	120.53 (19)	C25'—C26'—C21'	120.0
C17—C18—N4	123.1 (2)	C25'—C26'—H26'	120.0
C13—C18—N4	116.33 (15)	C21'—C26'—H26'	120.0
N2—C19—C12	107.93 (14)		
			
N1—C2—C3—C4	−179.6 (2)	C13—C12—C19—N2	−173.68 (16)
C7—C2—C3—C4	0.8 (3)	C10—C12—C19—C20	176.61 (18)
C2—C3—C4—C5	0.5 (3)	C13—C12—C19—C20	1.9 (3)
C3—C4—C5—C6	−0.7 (4)	N2—C19—C20—O1	29.5 (3)
C4—C5—C6—C7	−0.5 (3)	C12—C19—C20—O1	−145.7 (2)
C5—C6—C7—C2	1.7 (3)	N2—C19—C20—C21	−142.88 (17)
C5—C6—C7—C8	−179.35 (19)	C12—C19—C20—C21	41.8 (3)
N1—C2—C7—C6	178.41 (16)	N2—C19—C20—C21'	−152.33 (16)
C3—C2—C7—C6	−1.9 (3)	C12—C19—C20—C21'	32.4 (3)
N1—C2—C7—C8	−0.81 (19)	C8—C1—N1—C2	0.1 (2)
C3—C2—C7—C8	178.89 (16)	C3—C2—N1—C1	−179.22 (19)
N1—C1—C8—C7	−0.6 (2)	C7—C2—N1—C1	0.4 (2)
N1—C1—C8—C9	177.26 (15)	C10—C9—N2—C19	−0.33 (19)
C6—C7—C8—C1	−178.18 (19)	C8—C9—N2—C19	178.93 (15)
C2—C7—C8—C1	0.87 (19)	C12—C19—N2—C9	−0.45 (19)
C6—C7—C8—C9	4.0 (3)	C20—C19—N2—C9	−176.88 (15)
C2—C7—C8—C9	−176.98 (15)	C17—C18—N4—N5	3.8 (3)
C1—C8—C9—N2	−141.04 (18)	C13—C18—N4—N5	−177.93 (17)
C7—C8—C9—N2	36.4 (3)	C18—N4—N5—N6	170.4 (15)
C1—C8—C9—C10	38.0 (3)	O1—C20—C21—C22	−149.95 (13)
C7—C8—C9—C10	−144.54 (18)	C19—C20—C21—C22	22.5 (2)
N2—C9—C10—C12	0.97 (19)	C21'—C20—C21—C22	86.80 (6)
C8—C9—C10—C12	−178.21 (16)	O1—C20—C21—C26	25.4 (2)
N2—C9—C10—C11	177.12 (16)	C19—C20—C21—C26	−162.12 (12)
C8—C9—C10—C11	−2.1 (3)	C21'—C20—C21—C26	−97.82 (7)
C9—C10—C11—N3	−30 (11)	C26—C21—C22—C23	0.0
C12—C10—C11—N3	145 (11)	C20—C21—C22—C23	175.34 (13)
C9—C10—C12—C19	−1.23 (19)	C21—C22—C23—C24	0.0
C11—C10—C12—C19	−177.24 (16)	C22—C23—C24—C25	0.0
C9—C10—C12—C13	173.77 (16)	C23—C24—C25—C26	0.0
C11—C10—C12—C13	−2.2 (3)	C24—C25—C26—C21	0.0
C19—C12—C13—C14	−134.4 (2)	C22—C21—C26—C25	0.0
C10—C12—C13—C14	51.9 (2)	C20—C21—C26—C25	−175.43 (13)
C19—C12—C13—C18	49.9 (3)	O1—C20—C21'—C22'	−152.61 (14)
C10—C12—C13—C18	−123.87 (19)	C21—C20—C21'—C22'	−91.12 (7)
C18—C13—C14—C15	−1.0 (3)	C19—C20—C21'—C22'	29.3 (2)
C12—C13—C14—C15	−176.89 (18)	O1—C20—C21'—C26'	22.6 (2)
C13—C14—C15—C16	0.4 (3)	C21—C20—C21'—C26'	84.11 (7)
C14—C15—C16—C17	−0.1 (4)	C19—C20—C21'—C26'	−155.43 (12)
C15—C16—C17—C18	0.4 (4)	C26'—C21'—C22'—C23'	0.0
C16—C17—C18—C13	−1.1 (3)	C20—C21'—C22'—C23'	175.12 (12)
C16—C17—C18—N4	177.1 (2)	C21'—C22'—C23'—C24'	0.0
C14—C13—C18—C17	1.3 (3)	C22'—C23'—C24'—C25'	0.0
C12—C13—C18—C17	177.15 (17)	C23'—C24'—C25'—C26'	0.0
C14—C13—C18—N4	−176.97 (16)	C24'—C25'—C26'—C21'	0.0
C12—C13—C18—N4	−1.1 (2)	C22'—C21'—C26'—C25'	0.0
C10—C12—C19—N2	1.01 (19)	C20—C21'—C26'—C25'	−175.34 (12)

### Hydrogen-bond geometry (Å, º)

**Table:**

*D*—H···*A*	*D*—H	H···*A*	*D*···*A*	*D*—H···*A*
N2—H2*A*···O1^i^	0.92 (1)	1.95 (1)	2.8526 (17)	166 (2)
N1—H1*A*···N3^ii^	0.92 (1)	2.10 (1)	2.988 (2)	163 (2)

Symmetry codes: (i) −*x*, −*y*, −*z*+1; (ii) −*x*, −*y*−1, −*z*.
