# Supplementary figures and images for: Crystal structure of 4-(2-azido­phen­yl)-5-benzoyl-2-(1H-indol-3-yl)-1H-pyrrole-3-carbo­nitrile
Source: Acta Crystallogr E Crystallogr Commun. 2015 Apr 22;71(Pt 5):o335–6. doi: 10.1107/S2056989015006921 (PMC4420135; doi:10.1107/S2056989015006921)

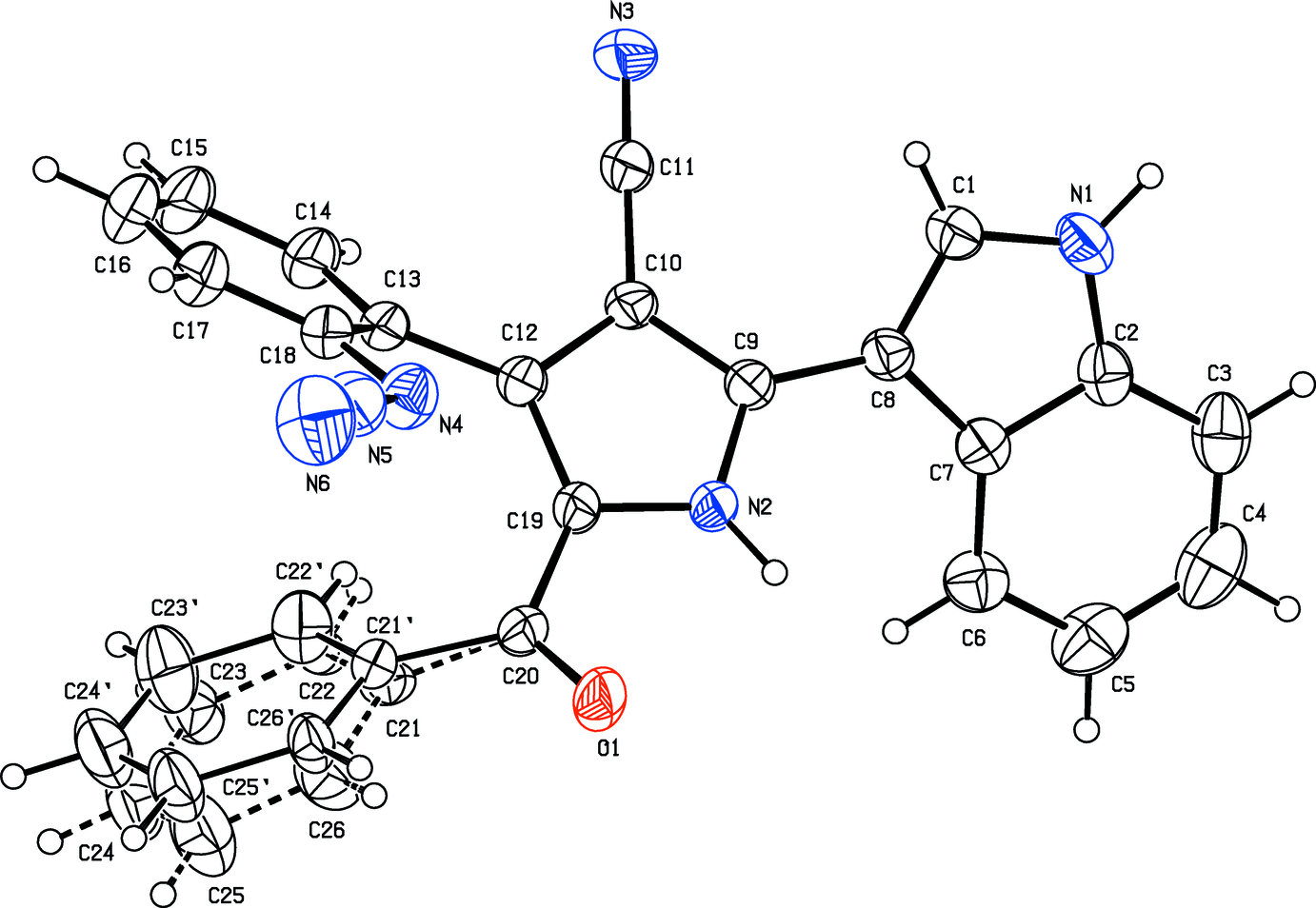

Supplement: Supplementary file 4 [file e-71-0o335-fig1.tif]

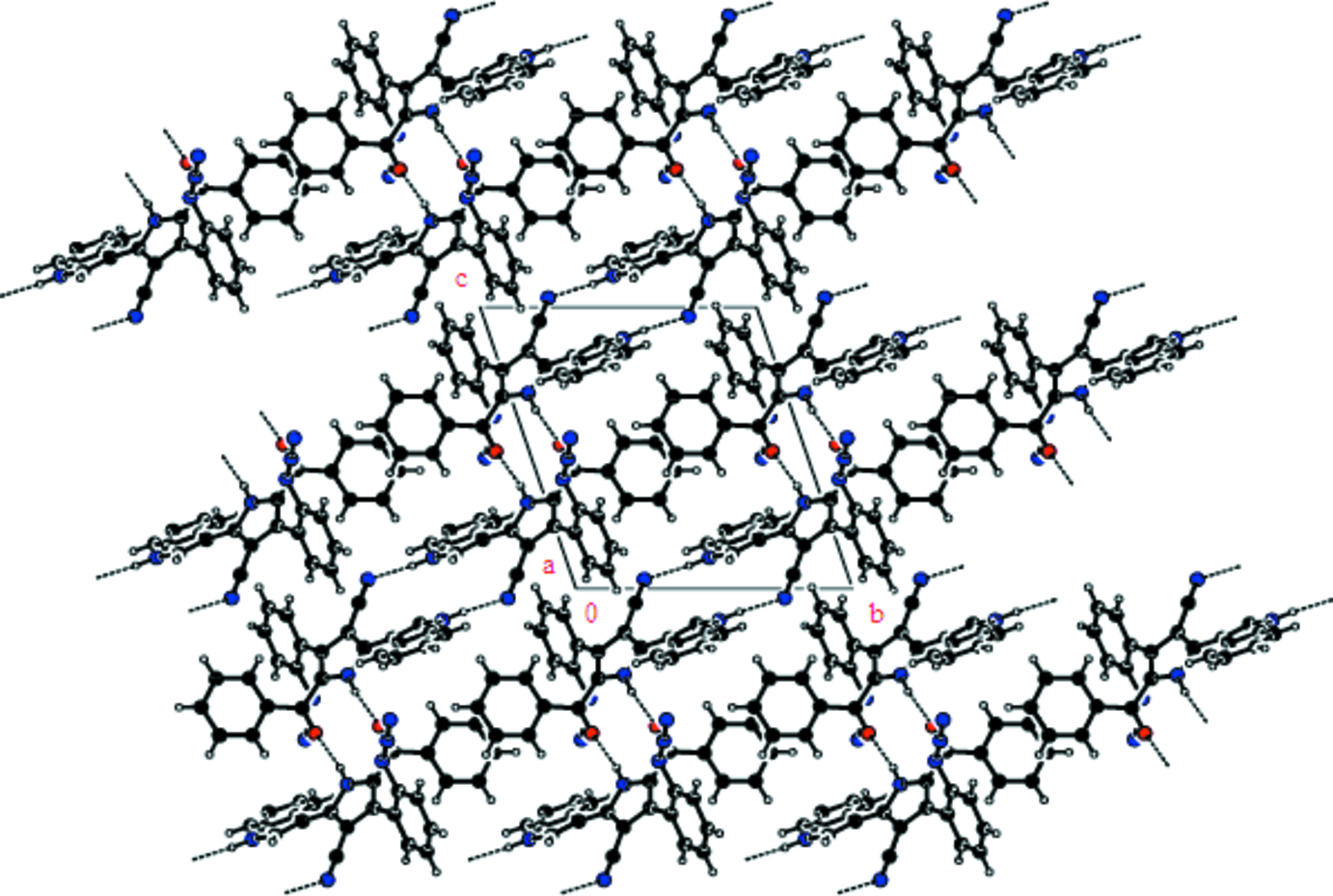

Supplement: Supplementary file 5 [file e-71-0o335-fig2.tif]
